# Impact of Wound Dressing on Mechanotransduction within Tissues of Chronic Wounds

**DOI:** 10.3390/biomedicines10123080

**Published:** 2022-11-30

**Authors:** Kelly McElvain, Joshua Klister, Alessandra Ebben, Sandeep Gopalakrishnan, Mahsa Dabagh

**Affiliations:** 1 Department of Biomedical Engineering, College of Engineering & Applied Science, University of Wisconsin-Milwaukee, 3200 N Cramer St., P.O. Box 784, Milwaukee, WI 53201, USA; 2 College of Nursing, University of Wisconsin-Milwaukee, 1921 E Hartford Ave., P.O. Box 412, Milwaukee, WI 53211, USA

**Keywords:** chronic wound, wound dressing, mechanotransduction, wound tissue, stiffness, computer modeling, tissue engineering

## Abstract

Chronic wounds are significant public health problems impacting the health-related quality of individuals’ lives (due to disability, decreased productivity, and loss of independence) and an immense economic burden to healthcare systems around the world. In this study, our main objective is to investigate how mechanotransduction can impact the healing process in chronic wounds. We have developed new three-dimensional models of wound tissue to study the distribution of forces within these tissues exerted by wound dressings with different characteristics. The roles of mechanical forces on wound healing have gained significant clinical attention; the application of mechanical forces is expected to influence the physiology of tissue surrounding a wound. We aim to investigate whether the force transmission within wound tissue is impacted by the dressing characteristics and whether this impact may differ with wound tissue’s properties. Our results show that wound dressings with lower stiffnesses promote force transmission within a wound tissue. This impact is even more significant on stiffer wound tissues. Furthermore, we show that size of wound dressing alters forces that transmit within the wound tissue where dressings with 9 cm length show higher stresses. The wound tissue stiffening has been associated with healing of a wound. Our results demonstrate that wounds with stiffer tissue experience higher stresses. Taken all together, our findings suggest that low stiffness of wound dressing and its size may be introduced as a criterion to explain parameters predisposing a chronic wound to heal. This study’s findings on the role of dressings and tissue characteristics demonstrate that precision dressings are required for wound management and understanding how a dressing impacts mechanotransduction in wound tissue will lead to design of novel dressings promoting healing in chronic wounds.

## 1. Introduction

Acute and chronic wounds are significant public health problems, impacting the health-related quality of individuals’ lives. Acute wounds result in over 17 million hospital visits annually in the United States. Chronic wounds are a significant socioeconomic burden on the healthcare system due to their high prevalence and recurrence. More than 1% of the population worldwide suffer from a chronic, nonhealing wound at some point in their lifetime. Annually, in the United States, 3 to 6 million chronic wounds occur, wherein the persistent inflammation of the wound bed alters mechanical properties, modulates cellular events, and ultimately delays healing [1,2,3]. Treatment of acute and chronic wounds creates an immense economic burden on healthcare systems globally. Treatment of chronic wounds in the United States alone has been conservatively estimated to cost $28 billion annually [4]. Commercially available wound dressings are extensively used as part of standard of care in wound management, yet there remains a lack of knowledge regarding the role of dressing characteristics on the wound healing process.

Wound healing is a complex and dynamic physiological process that consists of overlapping phases of coagulation, inflammation, proliferation, and tissue remodelling. These phases work to restore the tissue’s structural integrity after a wound occurs due to a disruption of the skin and underlying tissues followed by damage or insult [5,6,7]. Depending on the time required to heal, the wounds are classified as acute or chronic. Acute wounds often do not require extensive intervention and can heal on their own. However, chronic wounds may surpass the usual 8-to-12-week timeframe for healing, may not heal, or may be recurrent [8]. Therefore, chronic wounds require particular care and interventions to ensure the healing process is completed. As chronic wounds are persistent and may gradually become infected, they can cause pain, discomfort, hospitalization, and poor health-related quality of life for patients. This is especially common in diabetics and aged populations. Approximately 15% to 25% of diabetic patients develop a lower extremity ulcer at some point in their lifetime, while more than 10% of these cases lead to lower extremity amputations [2,9]. Thus, enhancing the understanding of the mechanotransduction mechanisms involved in nonhealing wounds in these patients is essential. Knowledge about the intrinsic and extrinsic force transmissions can assist in the design of more efficient and precision dressings for chronic wounds.

Several treatment methods have been applied for wound management including wound dressings, local injection of drugs mainly antibiotics, and in severe cases surgery. Among these more commonly used treatment strategies, dressing have caught more attention mainly because they are non-invasive and proven satisfactory success in promoting the healing of chronic wounds and preventing infection [1,4,5,6,10]. Furthermore, dressings are available in various materials and sizes allowing to match the body contour and ease their use at different areas of patients’ bodies by wound care teams or providers/caregivers [8]. Even though wound dressings are progressing, but there is a lack of literature evidence regarding how these dressings are impacting the mechanical force distribution in the wound microenvironment and how these forces contribute to the healing trajectory of a chronic wound. We are still far from having an efficient dressing that creates an optimal mechanical force in the wound bed to accelerate the healing process. Therefore, there is a need for new dressings which promote the healing process and take into account how mechanotransduction in wound tissue will be influenced by the dressing.

It has been previously shown that the response of a wound tissue to mechanical forces depends on the stiffness and biomechanical properties of the surrounding tissue [11,12,13,14,15,16,17,18,19,20,21]. This may vary with the anatomy of the patient, the anatomical location of the wound, and the patient’s health and comorbid conditions (if the patient either is elderly or has a history of chronic diseases such as diabetes) [5,8,22]. On the other hand, human skin is dynamically exposed to internal and external forces. Previous research suggested that specific wound dressings play a significant role in the prevention and reduction of pressure injury to those at risk. These studies concluded that patient outcomes may be improved by modifying pressure, friction, and shear forces along with quantifying the properties of the wound tissue and dressings used [23,24,25]. Furthermore, there is a pressing need for personalized dressings and to discern the mechanical forces involved in tissue alterations during the wound healing process. Therefore, without a clear identification of the forces and related mechanisms involved, the design of novel and patient-specific dressings to promote the healing of chronic wounds will remain challenging.

Recent studies have shown that applying mechanical forces can stimulate tissue regrowth and wound healing through mechanotransduction: a process in which mechanical stimuli applied to the tissue promotes a cellular response and improves tissue metabolism and cellular functions [1,4,5,6,23,24,26,27]. However, there remains a lack of literature evidence on how wound dressings influence mechanotransduction within a wound tissue. Past studies have demonstrated that tensile forces appear to stimulate tissue repair, whereas compressive forces appear to promote tissue destruction [5,28]. As the application of mechanical forces is expected to influence the physiology and structure of the tissue surrounding a wound, mechanotransduction has gained significant clinical attention [1,4,5,23,24,26,27,29,30]. Hence, understanding the mechanisms behind tissue repair and how they relate to mechanotransduction will help elucidate ways to treat chronic wounds more effectively. Precisely quantifying the forces involved in the wound healing process will determine the desired forces for effective healing which is fundamental.

Computational models have enhanced the understanding about the interplay of biological, chemical, and physical events during healing of a wound by providing quantities that are otherwise hard to determine [31,32,33]. These studies have applied either ordinary [32] or partial [33] differential equations (ODEs or PDEs, respectively) or with agent based models or developed a purely mechanical model including tissue growth or coupled basic biochemical fields with tissue nonlinear mechanics [31]. However, focus of all previous studies have been on modeling the complex tissue repair processes not on how a dressing may play role on tissue remodeling through mechanotransduction.

**Figure 1:**
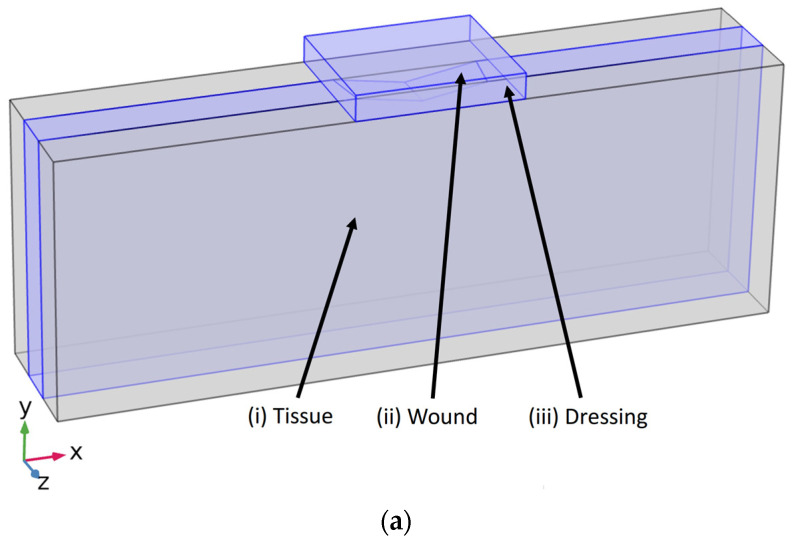

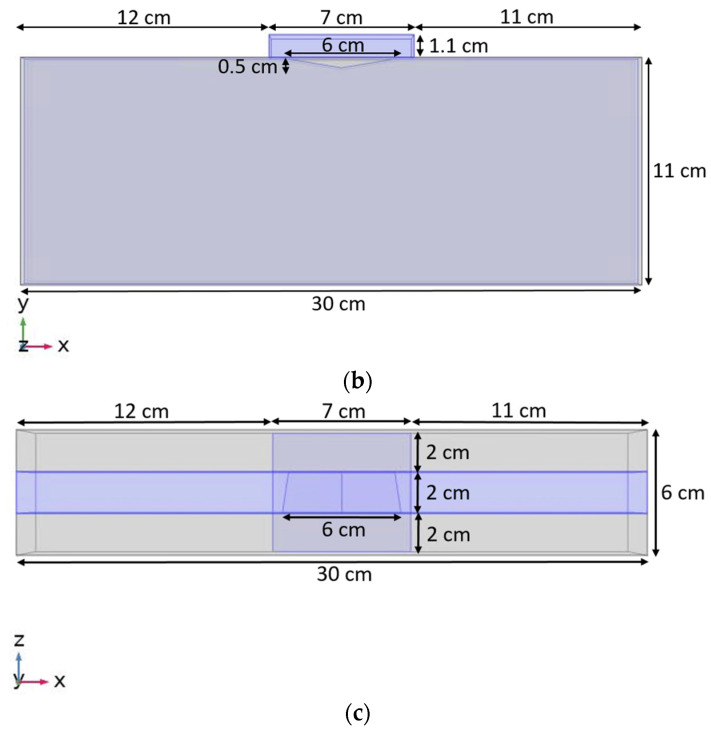
Model of an idealized 3-D chronic wound: (**a**) Components of the model including (i) tissue, (ii) wound, and (iii) dressing; (**b**) Frontal view of the geometry and components with dimensions; (**c**) Aerial view of the geometry and components with dimensions.

In the present study, we take first step to elucidate how application of a wound dressing and dressing’s characteristics will impact force transmission within a wound chronic wound. Distinct stiffnesses will be assigned to the wound tissue representing different condition (diabetics, aging, etc.) which may have led formation of a chronic wound. A three-dimensional computational model of a wound tissue under treatment is developed. As seen in Figure 1, wound dressing is placed on a wound tissue to transfer the force from the dressing throughout the wound tissue. We have quantified the force transmission along the wound tissue. We have studied the impact of both dressing and wound tissue to enhance our understanding of which variables have a key impact on force transmission. The knowledge gained is intended to help determine which available dressings are more effective in the wound healing process of patient-specific cases. In addition, findings of this study will assist the design of innovative, more effective wound dressings to promote the healing of chronic wounds.

## 2. Materials and Methods

In this study, our model of chronic wound under treatment consists of a homogeneous wound tissue and a wound dressing, depicted in Figure 1. The perspective view of our wound tissue model is depicted in Figure 1a and geometry of model components with dimensions are presented in Figure 1b. Figure 1c shows frontal view of the model with details about components.

Model geometry was generated by the finite element method solver via COMSOL MULTIPHYSICS v. 5.5 (COMSOL AB, Stockholm, Sweden). The computational results for von Mises stress are then determined and examined for mesh density independence. All components were assigned with material properties, mesh specifications, and boundary conditions (shown in Table 1). The wound tissue is 30 cm long (*x*-axis) by 11 cm tall (*y*-axis) by 6 cm wide (*z*-axis). The wound was cantered on the top of the tissue creating a thin concave triangular cavity. The wound’s dimensions were 0.5 cm at the maximum depth (*y*-axis) by 6 cm long (*x*-axis) by 2 cm wide (*z*-axis). The height and width of the dressing also remained constant in all studied cases. These dimensions were 1.1 cm tall (*y*-axis) by 6 cm wide (*z*-axis). The dressing width spanned the exact width of the tissue, with the wound cantered symmetrically beneath it (see Figure 1b,c). The dressing length (*x*-axis) was varied between 7 cm, 9 cm, and 11 cm, which is specified in each case described in Table 1. These dimensions were altered and analysed to observe, test, and quantify how the dispersing 4 kPa [34] load influences and affects the maximum force and the total von Mises stress (Pa) within and between the tissue and dressing. Utilizing COMSOL, the resulting load on the tissue model was simulated. The von Mises stress was used to measure the stress from an external load. To mimic biological dermis tissue, the deepest portion of the model (the side opposite of the dressing placement) was fixed. The external load was dispersed evenly on the superficial side of the dressing, bearing down toward the tissue. It was applied at 4 kPa throughout all trials of the simulation.

In this study, a range of properties was used to reflect various mechanical properties and makeups of both the wound tissue and dressing. The tissue was treated as an elastic material, with Young’s moduli of 50 kPa, 200 kPa, 1 MPa, and 100 MPa [35,36]. The dressing was treated as elastic material, with Young’s moduli of 10 kPa, 60 kPa, and 90 kPa [1]. These values were selected and simulated in accordance with the biological spectrum of stiffnesses of human skin. Table 1 demonstrates the properties applied in our studied cases.

The initial set of simulations was run to compare how dressing and wound tissue stiffness relate to extracellular matrix (ECM) stiffnesses. To do so, the various trials shown in Table 1 were run. This set of combinations of simulations resulted in 12 different trials. The dressing length remained at a constant 7 cm throughout these trials, controlling all properties except the ECM and dressing stiffness. The next set of simulations was run to compare how ECM stiffness and dressing length correlate. In these simulations, the dressing stiffness was cycled between 10 kPa, 60 kPa, and 90 kPa, with a 9 cm or 11 cm dressing length. This allowed for six trial combinations, which were then tested with various ECM stiffnesses of 50 kPa, 1 MPa, and 100 MPa. This set of combinations of simulations resulted in 18 different trials. As seen in Table 1, we studied 30 cases in total.

The data of domain coordination and von Mises stress were exported from COMSOL as four-column matrices into a four-column text file to be used in MATLAB. A code was created to sort specified sections within the XYZ model. This was completed to isolate and record the stress values at the most superficial part of the wound tissue at 11 cm in the simulation. With the height controlled, plots were formed to display how the von Mises stresses compare over the length of the tissue. Another code was generated in MATLAB to record the maximum stress in each trial, regardless of position within the model. The resulting data was plotted and analyzed.

## 3. Results

### 3.1. Impact of Dressing Stiffness and Tissue Stiffness on von Mises Stress within Wound Tissue

**Figure 2:**
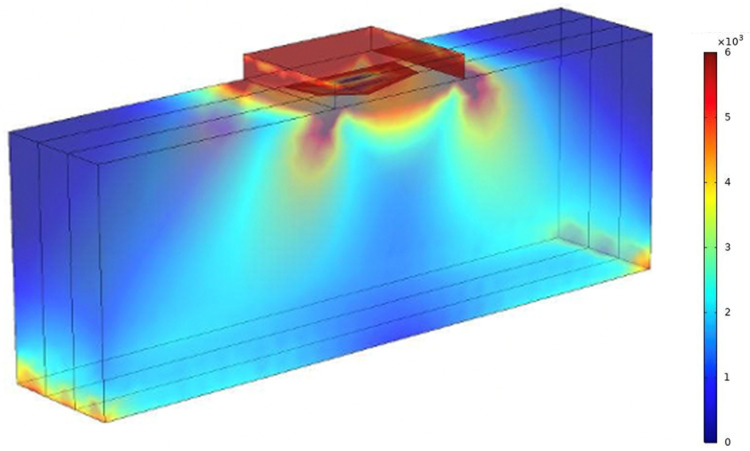
Distribution of von Mises stress (N/m^2^) magnitude on the dressing and wound tissue. The stiffness of the tissue and dressing are 10 kPa and 50 kPa, respectively.

**Figure 3:**
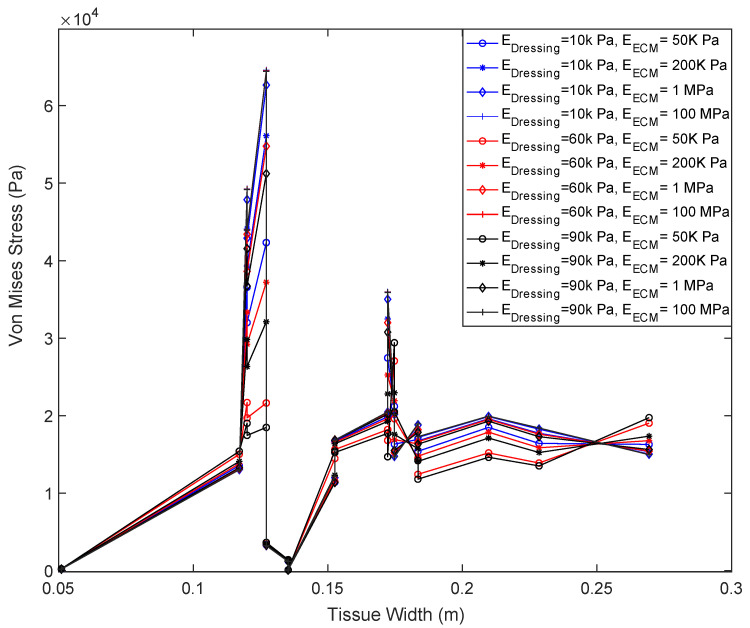
The von Mises stress values in accordance with the location on the tissue surface when dressing length is maintained at 7 cm.

Figure 2 demonstrates the distribution of von Mises stress within the dressing and wound tissue. The stiffness of the tissue and dressing are 10 kPa and 50 kPa, respectively. Figure 3 depicts the relationship between the dressing stiffness and wound tissue stiffness when the dressing size was held constant with a length of 7 cm. The trend in Figure 3 shows that ECM stiffness has significant and direct impact on the von Mises stress within the wound tissue in a way that as the ECM stiffness increases, the von Mises stress also increases. Additionally, Figure 3 demonstrates that the dressing stiffness and the von Mises stress within the wound tissue are inversely related; as the dressing stiffness increases, the von Mises stress decreases. As expected, the regions of the wound tissue adjacent to the dressing experience higher force transmission compared to the rest of wound tissue (12–18 cm below the wound-tissue interface).

### 3.2. Impact of Dressing Size on von Mises Stress within Wound Tissue

**Figure 4:**
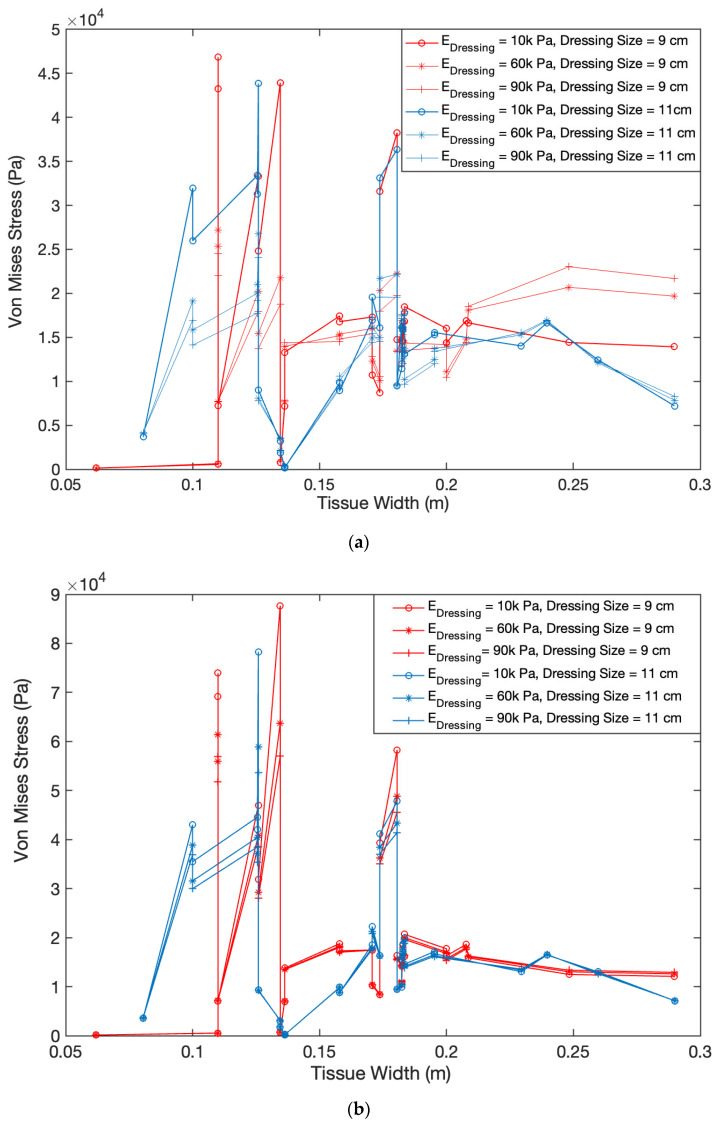

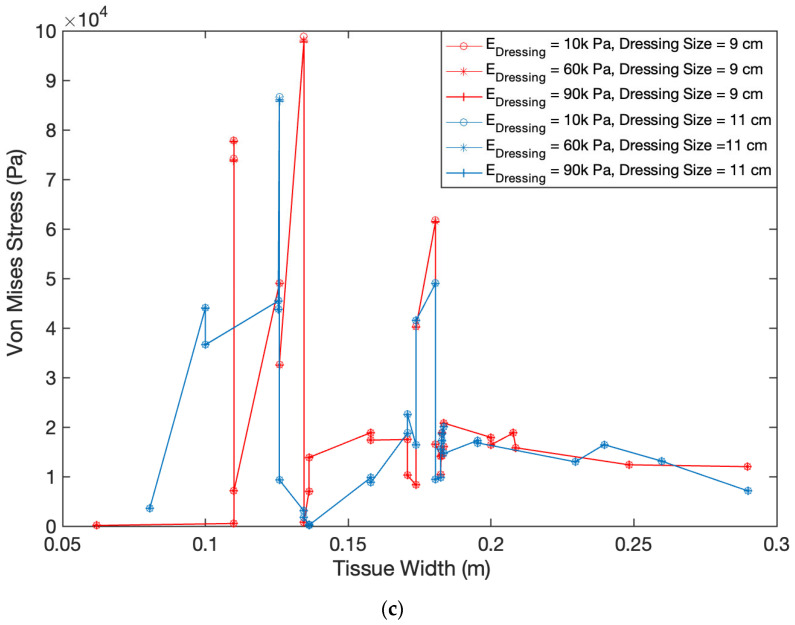

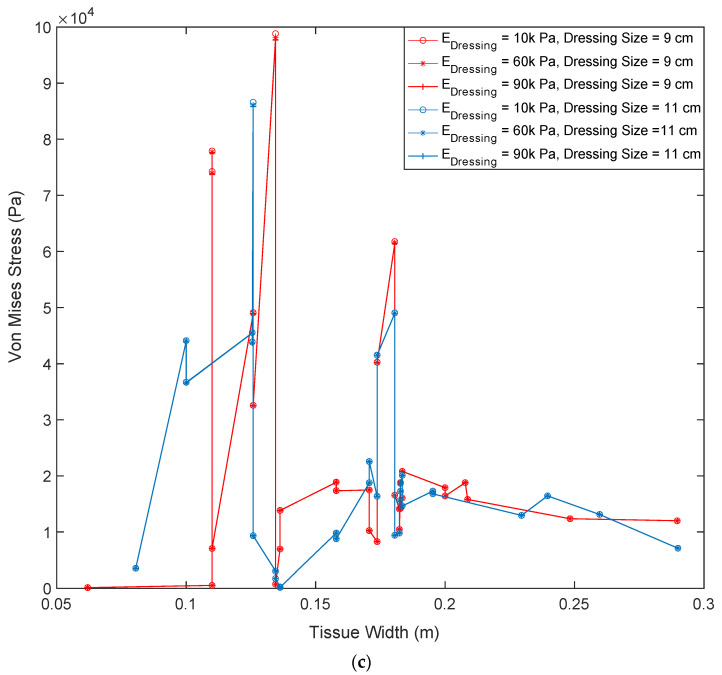
The von Mises stress values in accordance with the location on the tissue surface when dressing length is varied from 9 cm to 11 cm: (**a**) ECM stiffness is controlled at 50 KPa; (**b**) ECM stiffness is controlled at 1 MPa; (**c**) ECM stiffness is controlled at 100 MPa.

Figure 4 demonstrates how dressing size will alter the force transmission within a wound tissue. We also examined the impact of wound tissue’s stiffness in results shown in Figure 4 by looking at three different stiffness values of 50 kPa, 1 MPa, and 100 MPa. In Figure 4, within each plot, two dressing sizes of 9 cm and 11 cm are compared, while the dressing stiffness is ranged between 10 kPa, 60 kPa, and 90 kPa. It is observed that there are lower von Mises stress values when the larger 11 cm dressing is being used; this reflects an inversely proportional relationship between the dressing length and von Mises stress. By comparing Figure 4a–c, it is seen that the lower ECM stiffness results in a lower Von Mises stress. This reflects a directly proportional relationship between ECM stiffness and von Mises stress. Furthermore, this comparison shows that dressing stiffness does not impact the stress magnitude in either of the dressing sizes in stiffer wound tissues. Dressing size and properties have a significant impact when the wound tissue is soft. Figure 4 shows that softer dressings with a length of 9 cm exert higher stresses on the tissue beneath the dressing than a longer dressing.

### 3.3. Impact of Various Dressing and Tissue Properties on Maximum Force Transmission within Wound Tissue

**Figure 5:**
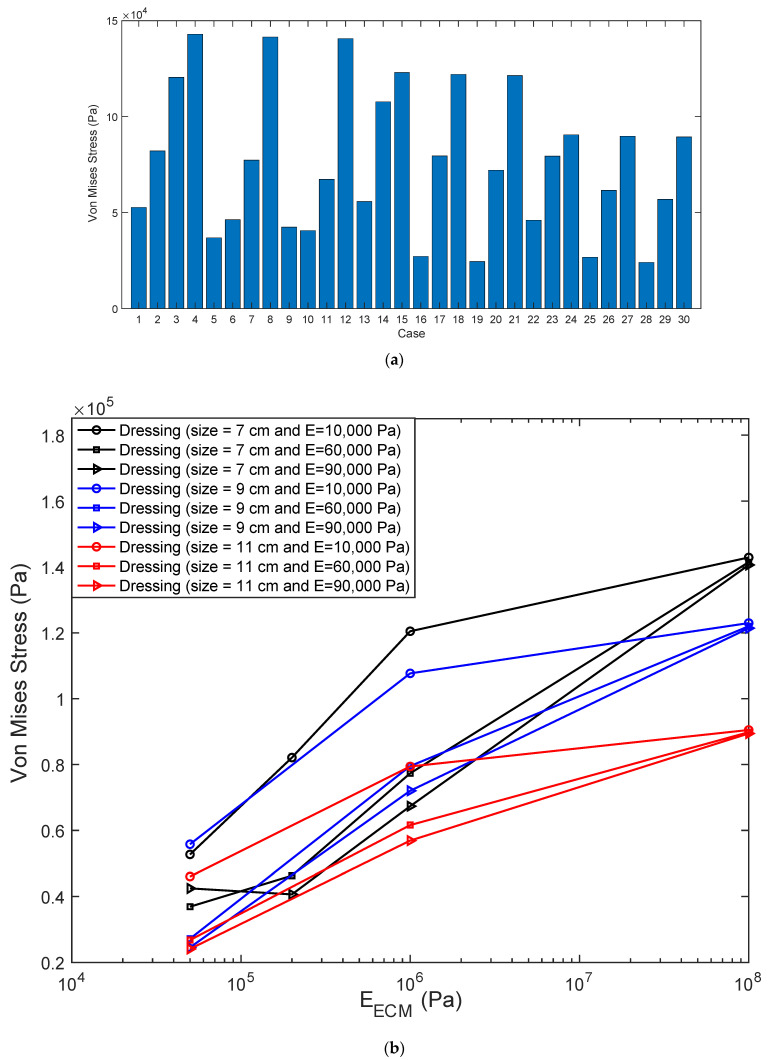
The maximum von Mises stress value within a wound tissue for each of the cases in Table 1. (**a**) Shown as histogram, (**b**) The maximum of von Mises stress within a wound tissue, treated by different dressings, is plotted against wound tissue’s stiffness.

Figure 5 shows the maximum magnitude of von Mises stress within the wound tissues. Figure 5a demonstrates the data in form of histogram of stresses within wound tissue for all of studied cases (presented in Table 1). Histogram represents the maximum magnitude of von Mises stresses within each tissue by a bin whose length shows the stress magnitude for that tissue. In cases 1 through 12, the dressing length was kept similar, so that the impact of dressing and ECM on force transmission can be clearly analyzed. In cases 1 through 4, the dressing stiffness remained the same, while the ECM stiffness increased. This demonstrates that the forces within tissue increase directly with each incremental increase in ECM stiffness. This relationship is repeated in cases 5 through 8, as well as cases 9 through 12. Moreover, Figure 5a depicts a directly proportional relationship between the dressing stiffness and the maximum force within the wound tissue: as the dressing stiffness increases, the maximum force on the wound tissue decreases. When the dressing size changes from 7 cm to 9 cm and 11 cm (cases 13 through 30), the stresses transmitted to ECM decreases independent of ECM and dressing stiffnesses. To analyze these data better, we plot the maximum magnitude of von Mises stress within the wound tissues against tissue stiffness in Figure 5b. With dressing becoming longer, the impact of its stiffness decreases but wound tissue’s stiffness still has significant impact with stiffer wound tissues experiencing higher stresses.

## 4. Discussion

In this study, our objective was to enhance our understanding of how characteristics of a wound dressing can impact force transmission within a wound tissue which may have distinct stiffnesses representing patients with different underlying diseases (diabetics, aging, etc.). The importance of our study is that chronic wounds continue to result in patient discomfort and poor quality of life. These wounds lead to tremendous economic and lifestyle burdens, considering hospital costs, disability, decreased productivity, and loss of independence. Therefore, it is crucially important to develop novel methods to improve current treatment strategies for chronic wounds. Commercially available wound dressings are extensively used as a part of the standard of care in wound management, but there is still a lack of knowledge regarding the mechanotransduction mechanisms by which the dressing impacts the wound healing process.

Mechanotransduction and its role in the progression of other diseases (e.g., atherosclerotic cardiovascular diseases and cancer) has been intensely studied [14,15,16,17,37,38,39,40,41,42]. Dabagh et al. [42,43,44] have studied mechanotransduction in endothelial cells to elucidate mechanisms involved in progression of atherosclerotic cardiovascular diseases and cancer metastasis. In this study, we take the first steps to understand how mechanotransduction can enhance our understanding of chronic wounds’ healing process and mechanisms underlying the healing of these wounds. Biological experiments and clinical trials have elucidated much of our current understanding of wound healing, however, with current experimental techniques it is difficult, or even impossible, to investigate many aspects of this complex process. To that end, mathematical models and theoretical descriptions have offered a way to explain and potentially predict certain wound healing behaviors in relation to mechanotransduction and other processes [11,12,13,14,15,16,17,18,19,20,21]. In this study, we have developed a 3D model of a chronic wound under treatment consisting of a homogeneous wound tissue and a wound dressing (Figure 1). We applied this model to investigate how dressing and tissue properties impact mechanotransduction within a wound tissue.

Our results show that stiffnesses of both wound dressings and wound tissue alter the distribution of von Mises stress at the interface of dressing and wound tissue (Figure 3 and Figure 4) and within wound tissue (Figure 5). As seen in Figure 3, when a shorter dressing with a length of 7 cm is inserted, the stiffness of ECM and dressing had opposite impact on the stresses experienced by wound tissue at its interface with the dressing. Stiffer ECM experience high stresses when treated with a softer dressing while the regions of the wound tissue adjacent to the dressing experience higher forces. With increasing the dressing’s length from 9 cm to 11 cm, the stresses at the interface of wound tissue and dressing drop significantly. ECM’s stiffness shows the same impact at dressing of 9 and 11 cm as seen at 7 cm with lower ECM stiffness resulting in a lower Von Mises stress. However, the dressing’s stiffness does not follow its behavior observed at length of 7cm. With increasing the length of dressing, the impact of its stiffness shrinks dramatically (Figure 4b). This impact is even more significant in stiffer ECM where dressing’s stiffness does not show any influence on stresses (Figure 4c).

To examine the impact of dressing’s stiffness and length on stresses transmitted to wound tissue, we quantified these stresses (Figure 5a,b). ECM stiffness shows similar influence on stresses with wound tissue as seen at the interface with the dressing. Stiffer ECM results in higher stresses. Furthermore, the stiffening of dressing leads to lower stresses within wound tissue as it was observed at the interface of tissue and dressing (Figure 3 and Figure 4). Changing the size of dressing from 7 cm to 9 cm and 11 cm led to lower transmission of stresses within wound tissue. An interesting result was seen in Figure 5b showing that dressing’s stiffness had significant impact on stresses in the wound tissue though this impact was higher at shorter dressing. This impact was not seen in the results of stresses at the interface of wound and dressing shown in Figure 4.

Taken altogether, our results show that lower dressing stiffness will cause higher force transmission to a wound tissue, independent of wound tissue’s stiffness. Size of dressing also appeared to alter the force transmission where based on our results the optimized length of a dressing to promote force transmission is 9 cm (see Figure 3 and Figure 4). Furthermore, we found that at the interface of the wound and the dressing, the greatest von Mises stresses were experienced. Alternatively, to increase the maximum force at the tissue-dressing interface in a stiffer ECM, the stiffness of the dressing can be decreased, and a smaller dressing length can be used. Balancing, further investigating, and testing these properties and parameters is important to consider when designing an ideal precision wound dressing for chronic wounds.

There were a few limitations of the present study. Firstly, the three-dimensional computational model did not fully represent the patient-specific complexity of a wound tissue. Another limitation is that the present study did apply mechanical properties for wound tissue from literature. For validation of computational model’s findings, initially in vivo experiments will be conducted, and animal’s tissue stiffness will be quantified using atomic force microscopy (AFM). Nonetheless, we have been able to gain important insights into the potential role of wound dressing on forces transmitted to a wound tissue. We have developed an investigational platform that can be used in future work. In future studies, when observing von Mises stress, the wound tissue should be considered anisotropic [45]. Continuing analysis of anisotropic tissue may provide significant data for soft tissue applications. This could provide new information that will change the desirable wound dressing properties for different types and locations of chronic wounds on patients. Additionally, the quantification of the properties of various tissue types, geometries, and localizations could lead to the development and design of a novel wound dressing that will efficiently, effectively, and comfortably provide treatment to patients. Future research should investigate a wider range of applications surrounding patient-specific tissue characteristics. Geometries should be simulated that mimic biological chronic wounds for further findings in relation to the impact of wound dressing stiffness on force transmission. Various dressing geometries, especially thicknesses, should be studied with different dimensions. This will help to relate the dressing and wound shape and size to force transmission and mechanotransduction in wounds. To further this research study, in vivo and in vitro studies will be conducted to support or disprove the current data collected from the mathematical models.

## 5. Conclusions

The present study has investigated the role of wound dressing characteristics on force transmission through chronic wound tissues with distinct properties. Our main objective is to take first step toward personalized wound dressings for patients who may have different medical histories leading to formation of chronic wounds. Our results showed that the dressing’s length and stiffness alter the stresses at the interface of dressing and tissue and within wound tissue. Stiffer wound tissues show higher response to stress transmission where the higher stresses are observed in response to softer and shorter dressings. Our findings suggest that the optimized length of a dressing to promote force transmission is 7–9 cm with stiffness of 10 KPa. Our study highlights important of patient-specific dressing characteristics. We have shown that through the analysis of wound dressing characteristics and tissue stiffness, the distribution of force transmission within the tissue can facilitate the acceleration of novel developments to heal chronic wounds.

## Figures and Tables

**Table 1:** Description of cases with the corresponding model values given 4000 N/m^2^ force exerted on top of the dressing.

Case	Dressing Size (cm)	Dressing Stiffness (Pa)	ECM-Stiffness (Pa)
1	7	10,000	50,000
2	7	10,000	200,000
3	7	10,000	1,000,000
4	7	10,000	100 × 10^6^
5	7	60,000	50,000
6	7	60,000	200,000
7	7	60,000	1,000,000
8	7	60,000	100 × 10^6^
9	7	90,000	50,000
10	7	90,000	200,000
11	7	90,000	1,000,000
12	7	90,000	100 × 10^6^
13	9	10,000	50,000
14	9	10,000	1,000,000
15	9	10,000	100 × 10^6^
16	9	60,000	50,000
17	9	60,000	1,000,000
18	9	60,000	100 × 10^6^
19	9	90,000	50,000
20	9	90,000	1,000,000
21	9	90,000	100 × 10^6^
22	11	10,000	50,000
23	11	10,000	1,000,000
24	11	10,000	100 × 10^6^
25	11	60,000	50,000
26	11	60,000	1,000,000
27	11	60,000	100 × 10^6^
28	11	90,000	50,000
29	11	90,000	1,000,000
30	11	90,000	100 × 10^6^

## Data Availability

Data will be available at: https://panthers-my.sharepoint.com/personal/dabaghme_uwm_edu/_layouts/15/onedrive.aspx?id=%2Fpersonal%2Fdabaghme%5Fuwm%5Fedu%2FDocuments%2FDabagh%20Lab%2FStudents%2FKelly%2FWound%20Healing%2FImpact%20of%20Wound%20Dressing%20on%20Mechanotransduction%20within%20Tissues%20of%20Chronic%20Wounds%2FCases (accessed on 28 November 2022).
